# Mapping the Intersecting Contexts of Migration and Pediatric Pain over the Last Decade: A Rapid Scoping Review Protocol

**DOI:** 10.3390/children12101325

**Published:** 2025-10-02

**Authors:** Mica Gabrielle Marbil, Josep Roman-Juan, Megan MacNeil, Sean Lindsay, Diane Lorenzetti, Melanie Noel, Kathryn A. Birnie

**Affiliations:** 1 Department of Psychology, University of Calgary, Calgary, AB T2N 1N4, Canada; mica.marbil@ucalgary.ca (M.G.M.); sean.lindsay1@ucalgary.ca (S.L.); melanie.noel@ucalgary.ca (M.N.); kathryn.birnie@ucalgary.ca (K.A.B.); 2 Alberta Children’s Hospital Research Institute, University of Calgary, Calgary, AB T2N 4N1, Canada; 3 School of Public Health, University of Alberta, Edmonton, AB T6G 2R3, Canada; megan.macneil@ucalgary.ca; 4 Department of Anesthesiology, Perioperative, and Pain Medicine, University of Calgary, Calgary, AB T2N 1N4, Canada; 5 Chronic Pain Network, McMaster University, Hamilton, ON L8S 4L8, Canada; 6 Health Sciences Library, Department of Community Health Sciences, University of Calgary, Calgary, AB T2N 1N4, Canada; dllorenz@ucalgary.ca

**Keywords:** health equity, social indicator of health, migration, pediatric pain, rapid review, scoping review

## Abstract

**Highlights:**

**What are the main findings?**

- Results of this rapid scoping review are expected to summarize what is known and what gaps remain in our knowledge of pediatric chronic pain among migrant youth on a global scale.

**What is the implication of the main finding?**

- Sociocultural contexts influencing pediatric chronic pain among migrant youth will be highlighted, offering insights into systemic influences on pediatric chronic pain development and potential targets for intervention.

**Abstract:**

Background/Objectives: Migrant youth often experience multiple, intersecting systems of oppression (e.g., racism, poverty, and discrimination) that may contribute to disparities in pediatric pain prevalence, severity, and management. However, pain in migrant youth remains poorly understood. This rapid scoping review will examine the nature and extent of the existing literature on pain among migrant youth. Methods: This protocol has been preregistered on the Open Science Framework. The review will follow guidelines for conducting and reporting rapid and scoping reviews, and will be guided by PCC (population, concept, context) and PROGRESS-Plus methodological frameworks. Electronic searches will be conducted in MEDLINE, CINAHL, and Scopus for primary research studies published since 2015 that describe and examine pain among migrant youth (age < 18 years). Two reviewers will independently screen titles, abstracts, and full texts, with disagreements resolved by consensus or a third reviewer. Data charting will be piloted on 5–10 studies, then independently conducted by two reviewers. Extracted data will include study characteristics (authors, year, purpose, methodology); participant sociodemographic information (e.g., racial and/or ethnic identity, age, sex, gender identity, sexual orientation, socioeconomic position); migration status; countries of origin and destination; definition and characteristics of pain; and measures of systemic factors (e.g., racism). Results: Findings will be synthesized descriptively and interpreted within sociocultural and geopolitical contexts to better understand pain among migrant youth. Conclusions: This review will aim to provide critical insights into the intersections between migration and pediatric pain, offering guidance for future research, clinical practice, and policy to improve pain management and outcomes for migrant youth.

## 1. Introduction

Pediatric pain is a multifaceted health issue influenced by biological, psychological, and social factors [1]. Acute pain is common in youth [2], with up to 96% of schoolchildren experiencing acute pain in one month [3]. If not adequately managed, acute pain can persist past its functional utility and have potential long-term impacts [4,5], including the development of chronic pain [6]. Chronic pain (i.e., pain > 3 months [7]) affects one in five youth [8] and is associated with worse functioning, quality of life, and mental health, including into adulthood [9,10,11].

Pain relief is a fundamental human right [12]. However, equity-denied groups (e.g., racialized, lower socioeconomic position) are both disproportionately affected by pain and underrepresented in pain research [13]. Among them, migrant youth may experience unique vulnerabilities that exacerbate disparities in pain prevalence and management. Despite increased global migration [14], migrant youth remain underrepresented in pediatric pain research [15], contributing to a critical gap in our understanding of pain disparities and hindering the development of inclusive care models.

Migration status is a key social indicator of health [16,17]. While migration varies between immigrants, refugees, and asylum-seekers, migrants are often subject to challenges and oppression in destination countries, including poverty, racism, xenophobia, and limited access to health care [18]. In turn, these structural and social barriers can have profound effects on migrant health [19]. Moreover, migration may differentially impact adults and youth, such that the experiences of migrant parents may also influence the health of their children [20,21]. The minimal research that exists suggests that both migration background [22] and anti-immigration attitudes [23] are associated with increased risk of pediatric chronic pain. Still, much of pediatric pain research overlooks the intersection of migration with other individual (e.g., age, sex [biological category assigned at birth], gender identity [socially constructed and embodied roles and identities], sexual orientation, ethnic and/or racial identity, socioeconomic position) and social factors (e.g., culture, religion, family dynamics), as well as policies and oppression (e.g., income inequality, discrimination) in destination countries, which can interact to influence health [21,24].

In recent years, there has been a rise in discriminatory policies and rhetoric targeting migrants [25], along with pervasive negative media portrayals [26] and disinformation around migrants and migration [27]. These can compound oppression (e.g., racism [28]) that influences pain. It is necessary to normalize migration narratives [14] and enhance our understanding of how migration and associated social processes affect health in order to address pain disparities. A rapid synthesis of the literature can help to elucidate the association between migration and pediatric pain and identify knowledge gaps to inform future research, clinical practice, and policy interventions. To date, there are no systematic or scoping reviews focused on migration and pediatric pain, despite evidence that migrant children and youth are at higher risk of poor health outcomes (e.g., mental health [29]). Further, while some reviews have investigated the health of migrant youth more broadly [21,30,31,32], pain as a specific outcome is rarely examined and addressed.

Given the urgent need to inform clinical and policy responses to health disparities in pediatric migrant populations, we will conduct a rapid scoping review to understand what is known about pain in migrant youth and explore intersecting contexts therein (e.g., race, socioeconomic position). Rapid reviews require a streamlined and systematic approach to synthesize evidence in a timely and resource-efficient manner, particularly in emerging areas where comprehensive reviews may be limited in scope or feasibility [33]. We aim to synthesize evidence on migration and pediatric pain, examine how pain and migration are conceptualized and defined in the literature, and identify knowledge gaps. We also aim to provide guidance on conducting more inclusive research [34] by framing our findings and discussions within broader sociocultural and structural systems and contexts. In so doing, we are responding to goals and priorities for increased equity in pain care [4,35] and calls for anti-racist and anti-oppressive pain research [36].

## 2. Materials and Methods

The protocol for this review has been registered on the Open Science Framework [37].

This rapid scoping review will follow the methodological guidance from the World Health Organization (WHO [38]) and Cochrane [33,39,40,41] for rapid reviews. Additionally, scoping review methodology (e.g., Arksey and O’Malley’s seminal framework [42], with Peters et al.’s updated recommendations for the Joanna Briggs Institute [43]) will be integrated to improve methodological rigor. The review will be reported in accordance with the Preferred Reporting Items for Systematic Reviews and Meta-Analyses Extension for Scoping Reviews (PRISMA-ScR) [44].

Alongside this, we will also use the PROGRESS-Plus framework. PROGRESS-Plus captures social indicators of health, such as place of residence, racial and/or ethnic identity, occupation, gender, socioeconomic position [45], and other intersecting factors (e.g., sexual orientation), thus facilitating examination of the intersections between migration and pediatric pain.

We also intentionally adopt more inclusive language (e.g., from determinants to indicators of health) in this work, in line with recommendations for anti-racism in research [46]. We recognize that language is evolving and context-specific, and we strive to ensure our usage of language and knowledge is reflective of our commitment to anti-racism and anti-oppression.

### 2.1. Eligibility Criteria

Eligibility criteria were collaboratively developed and refined through pilot searches, guided by frameworks and recommendations for incorporating intersectionality in pain [47] and child health research [24].

Eligible studies will describe migrant children and adolescents (i.e., aged < 18 years, according to the United Nations’ definition for “children” [48]). We will adopt an inclusive definition of “migrant,” considering studies that describe (i.e., versus exclusively sample) first- (i.e., not born in the destination country) and second-generation (i.e., with at least one parent born outside of that country) migrant youth (e.g., immigrants, refugees, asylum-seekers), based on extant definitions and the literature. The United Nations Recommendations on Statistics of International Migration define “international migrant” as “any person who has changed his or her country of usual residence”, encompassing immigrants, refugees, asylum-seekers, and other individuals fitting this definition [14] (p. 21). However, research has shown differences associated with migrant generations (i.e., between first- and second-generation) and social contexts (e.g., parental functioning) on migrant youth health [21] and pediatric pain experiences [49]. Using a broader definition of “migrant” will enable the exploration of diverse migration experiences (e.g., forced displacement, voluntary migration, and asylum-seeking) and their association with pediatric pain. When interpreting the results of an included study, we will use the term for migration status included therein. Studies that do not allow for conclusions specific to migrant populations (i.e., migrants are not disaggregated in analyses), or that describe non-migrant populations or adult migrants (without youth), will be excluded.

This review will include peer-reviewed journal articles and studies examining or involving any type of pain (i.e., acute or chronic) and its related factors (e.g., prevalence, severity, management, and associated health outcomes). Any primary research study designs will be eligible, including quantitative, qualitative, and mixed-methods (cross-sectional, cohort, case–control, and intervention studies). We will exclude studies that do not examine pain or related health outcomes, as well as the grey literature, conference abstracts, book/chapters, case studies, commentaries, guidelines, and other non-clinical reports [33,50].

Only studies published within the last 10 years (i.e., since 2015) will be included. This timeframe is justified for two reasons: first, migration was included in the United Nations’ 2030 Sustainable Development goals in 2015; second, since then, several significant events (e.g., the rise of anti-migration sentiment and policies, the COVID-19 pandemic) have impacted migration and international travel [14]. This date restriction will ensure that the included evidence is recent and relevant, as is consistent with rapid review methodology. We will not limit the geographical scope, recognizing the global spread of migration beyond high-income countries and varying reasons for migration [14]. However, we will continuously refer to geographical contexts of studies to facilitate the consideration of health and health care disparities in migrant contexts.

Only studies published in English will be included, as this is the common language of the study team. Of note, however, language restrictions will only apply at study selection and not in searching, in line with rapid review recommendations [33]. Although most databases we will search primarily index English-language journals, this approach will allow us to identify non-English publications and document their number, providing transparency about the potential scope of excluded literature. Prior research has demonstrated that limiting to English-language publications does not impact the results of medical systematic reviews, but only for conventional (i.e., not alternative or complementary) medicine [51]. Given the global nature of migration and its potential differential influences on health based on geopolitical context, we acknowledge that relevant research may exist in other languages, and language bias can perpetuate inequities in health research [52]. While our approach cannot eliminate this bias, reporting on the number of non-English publications identified will help contextualize the impact of our language restriction.

### 2.2. Information Sources and Search Strategy

The search strategy has been developed collaboratively by the study team, which includes pediatric pain psychology and public health trainees, researchers, and a research librarian, with lived experiences of migration and experience in conducting systematic and scoping reviews. The electronic databases MEDLINE, CINAHL, and Scopus will be searched via Ovid, EBSCO, and Elsevier, respectively. These databases were selected to include coverage of the relevant literature across medical, allied health, and social science disciplines, balanced with a streamlined approach consistent with rapid review methodology. Posts will also be sent to pediatric pain and child health listservs (Pediatric Pain, Pain in Child Health, American Psychological Association [APA] Division 54: Society of Pediatric Psychology) to gather relevant articles not captured by database searches.

The preliminary search terms were derived from examination of existing reviews in pediatric pain [53] and migrant youth health [21], preliminary database searches, and pre-identified seed papers (i.e., papers meeting criteria for inclusion in the review, determined through discussion with the study team; see [22,54,55]). Final search terms will be tested and refined through an iterative search strategy development process.

As justified above, the search will be restricted to studies published from 2015 onward. Search terms will include keywords related to “pediatric pain,” “migration,” “migrant”, “immigrant,” “refugee,” and “asylum-seeker”, searched within relevant database-specific subject headings and in titles and abstracts.

Search results will be uploaded to and de-duplicated in Covidence [56], a review software program, to facilitate screening processes.

### 2.3. Study Selection and Screening

Study selection and screening will be guided by the eligibility criteria outlined above. Initial screening of titles and abstracts will be independently conducted by two members of the study team (M.G.M. and J.R.-J.) to identify potentially eligible studies. Prior to the screening process, a pilot exercise will be conducted using a subset of articles to ensure consistency in the application of eligibility criteria. As needed, discussions will be scheduled to resolve any issues related to the interpretation of criteria throughout screening.

During title and abstract screening, each article will be independently assessed by two reviewers for eligibility. Discrepancies in screening decisions will be resolved through discussion among reviewers. If consensus cannot be reached, a senior team member (K.A.B. and/or M.N.) will be consulted to make the final decision. If a decision on an abstract still cannot be made, the study will be included in a full-text review.

Subsequently, a pilot exercise for full-text screening will be performed with a subset of full-text articles to ensure consistency and accuracy in the application of eligibility criteria. The full text of each article will be independently assessed by two reviewers for inclusion. Any discrepancies in full-text screening decisions will be resolved through discussion among the reviewers, with a senior team member reviewer (K.A.B. and/or M.N.) available for consultation if needed. Unclear information in full-text articles will be addressed by contacting study authors for clarification, as required.

### 2.4. Data Charting/Collection/Extraction

The data charting form will be co-developed by members of the study team and finalized by consensus. This form will be developed based on PROGRESS-Plus factors [45], intersectional frameworks, and guidance on improving inclusivity in pain and child health research [24,34], as well as prior reviews on immigrant youth health [21]. The data charting form will be piloted on a sample of 5–10 studies to refine the extraction fields. As needed, consultation will be sought with additional experts in the fields of pediatric pain, migration, or intersectionality.

Data items to be collected include: author name(s), year of study, purpose, methodology, sampling, sample size, participant type (e.g., youth, parent, health professional, etc.) and sociodemographic information (e.g., sex, gender identity, age, sexual orientation, racial identity, ethnicity), reason for migration, migrant generation (i.e., first or second), countries of origin and destination, primary findings, type of pain (e.g., acute or chronic), pain outcomes and/or interventions (as applicable), and measures pertaining to systemic and sociocultural factors and mechanisms (e.g., discrimination).

Rules and procedures for the charting of each data item will be outlined in a detailed data charting protocol. Procedures for data charting of specific publication types will also be documented therein; for example, information for a single study reported in multiple publications will be extracted separately before being collated [57]. The protocol will be accessible to all team members to ensure consistency and accuracy in data charting.

Data charting will be performed by two independent reviewers using the standardized form developed. Discrepancies in extracted data will be resolved through discussion between the reviewers. If consensus cannot be reached, a third reviewer will be consulted to make the final decision. Unclear or missing information will be addressed by consulting the full text of the article or seeking additional expert opinion if necessary. The software used for managing the data extraction process will be Covidence.

### 2.5. Synthesis and Presentation of Results

Data extracted from included studies will be reviewed for completeness and correctness. Any discrepancies or errors identified during this review will be addressed by cross-checking with the original sources. Missing or unclear information will be resolved by consulting the articles’ full texts or seeking expert opinion if necessary.

A PRISMA flow diagram [44] will be used to document the study selection process. This diagram will illustrate the number of studies identified, screened, assessed for eligibility, and included in the review, providing a transparent and systematic overview of the selection process.

Per rapid review recommendations [33], data synthesis will involve descriptive summary and narrative interpretation of the characteristics and findings of the included studies. Results will be presented using tables, figures, and diagrams. Tables will summarize key characteristics and findings of the included studies, while figures and diagrams will illustrate patterns and relationships in the data. For example, a table may list the prevalence of pain among migrant youth across different studies, while a figure may show the distribution of pain severity by immigration status. To understand how pain in migrants has been understood in the literature, tables and figures are also planned to represent where knowledge exists and where gaps remain. Table 1 serves as an example of how data items may be presented in results syntheses:

Importantly, findings will be mapped onto intersectional frameworks for pain [47] and child health research [24] and interpreted within their sociocultural contexts (e.g., events surrounding the year of publication and location of the study) to highlight intersecting social and geopolitical factors shaping pediatric pain in migrant populations. This also enacts recommendations to shift from a disparity- to diversity-based framework by acknowledging societal and structural mechanisms that may impact pain, particularly in groups who are systemically oppressed [34]. We anticipate that this will be shown in a timeline via tabular and/or graphical representation of studies overlaying a chronological listing of significant geopolitical events impacting migration. Table 2 shows what this may look like in the final manuscript.

### 2.6. Ethics and Dissemination

Ethics approval for this rapid scoping review is not required at the authors’ institutions, as it will synthesize data from publicly available sources and previously published studies. No primary data collection or interaction with human participants will be conducted. However, given the importance of involving people with lived experience of a phenomenon in research that aims to describe them, we will engage at least one person with lived experience of pediatric pain and migration as a research team member, following guidelines on engagement in rapid reviews and fair compensation [58,59].

The findings of this review will be disseminated through multiple channels to maximize impact and reach. A peer-reviewed manuscript will be submitted. Additionally, results will be shared at relevant conferences with audiences of patients, researchers, health professionals, and/or policymakers, and with key interest holders, including organizations focused on pain, child health, and migrant well-being. Other knowledge mobilization activities (e.g., lay summaries, infographics) will be developed to enhance accessibility for non-academic audiences, particularly community organizations working with migrant youth. Where possible, findings will also be presented to patients and advocacy groups to ensure that lived experiences inform future research and policy initiatives and that generated knowledge reaches those whom it aims to describe and help the most.

## 3. Discussion

This rapid scoping review protocol outlines a systematic and equity-informed approach to mapping the literature on pediatric pain in migrant youth: a critically underexplored topic with growing relevance in global health. The review seeks to synthesize existing evidence while recognizing the complex sociocultural and geopolitical contexts that shape migrant health and pain experiences.

The urgency of this work is underscored by the global rise in migration [14], along with anti-migration sentiment and rhetoric [25], and persistent inequities in pain care faced by marginalized populations [13], including racialized youth [60,61] and the youth of lower socioeconomic positions [62]. Migrant youth often navigate overlapping systems of oppression—including racism, poverty, and discrimination—that may exacerbate pain experiences and restrict access to appropriate care. Yet, pediatric pain research rarely disaggregates data by migration status or simultaneously considers intersecting sociodemographic characteristics such as ethnic or racial identity, gender, and socioeconomic position. This review will address these gaps to identify how pain is defined, measured, and addressed in studies involving migrant youth, and highlight where knowledge remains scarce.

Importantly, this review also responds to calls for anti-racist and anti-oppressive research practices in pain science. By adopting inclusive definitions of migration and attending to structural mechanisms such as policy and discrimination, the review moves beyond deficit-based models to promote a more nuanced and context-sensitive understanding of diversity in pediatric pain. Planned synthesis strategies, including visual mapping of study characteristics and timelines overlaid with geopolitical events, will provide a multidimensional view of how migration and pain intersect across settings and populations.

While this rapid scoping review is designed to provide a timely and equity-oriented synthesis, several limitations must be acknowledged. First, the rapid review format necessitates methodological streamlining, including restrictions on publication date (2015 onward), language (English only at the study selection stage), and database scope (only three databases). Though consistent with rapid review guidance [33], these parameters may result in the exclusion of relevant studies published in other languages or earlier time periods, particularly from regions with extensive migration histories.

Second, the review will include only peer-reviewed primary research, thereby excluding the grey literature, community-based reports, and policy documents that could offer critical insights into the lived experiences of migrant youth. Given the underrepresentation of migrant populations in the academic literature, this exclusion may inadvertently reinforce existing knowledge gaps. However, this lack of non-peer-reviewed research may also serve as a call to action for more rigorous and focused research in this area. Supplementary efforts, such as outreach through pediatric pain and child health listservs, may help identify additional relevant studies, as recommended by some knowledge synthesis approaches; still, we acknowledge that the use of listserv postings may introduce some selection bias. Nonetheless, the review remains primarily anchored in indexed, peer-reviewed studies accessible through the selected databases.

Third, the synthesis will depend on how the included studies conceptualize and categorize both migration and pain. Variability in definitions (e.g., first- vs. second-generation migrants, voluntary vs. forced migration) and pain measurements (e.g., acute vs. chronic, self-reported vs. clinical assessments) may introduce heterogeneity that complicates comparison across studies. Moreover, many studies may not disaggregate findings by migration status or intersecting factors, limiting the review’s ability to explore related sociodemographic and systemic differences in pain experiences. Nevertheless, heterogeneity may also introduce necessary nuance to our own understanding of migration and pediatric pain, allowing for the integration of divergent perspectives in our conclusions.

Fourth, while our review will note the diversity of psychological and biological assessment methods reported, a detailed appraisal of these tools is outside the scope of our synthesis. Similarly, multimodal approaches integrating pharmacologic, physical, and psychosocial interventions are important aspects of pediatric pain care but fall beyond the remit of this rapid scoping review. Future research should aim to systematically evaluate the way psychological (e.g., anxiety and depression scales, pain catastrophizing), biological (e.g., neuroimaging, quantitative sensory testing), and pharmacological assessments and treatments are applied in pediatric pain studies involving migrant youth. Additionally, exploring adaptations of diagnostic and management approaches to serve diverse populations will be critical to advancing equitable, effective, and holistic pain care.

Finally, although this review incorporates intersectional and equity-oriented frameworks, its interpretive depth will ultimately be shaped by the extent to which the included studies themselves engage with such perspectives. A lack of anti-oppressive or culturally responsive methodologies within the literature may constrain the ability to fully assess structural influences on pain. Consequently, however, the review may illuminate not only gaps in knowledge but also gaps in research approach, reinforcing the need for future studies that are inclusive, equity-driven, and structurally informed.

## 4. Conclusions

This rapid review will provide a timely and critical synthesis of what is known about pediatric pain in migrant youth and their families, framed within the intersections of migration and other sociocultural and geopolitical dimensions of health. By addressing the lack of comprehensive reviews on this topic, we aim to contribute to a broader understanding of migration and pediatric pain and further inclusive and equity-driven research. We endeavor to use our privilege as researchers and clinicians to advocate for systemic change by identifying gaps in the literature and highlighting implications for clinical practice and policy to reduce health disparities and advance equitable pain care for migrant youth and their families.

## Figures and Tables

**Table 1:** Sample data synthesis presentation.

Study	Study Characteristics				
Author(s), year, country(ies)	Study objective(s)	Study type and methodology	Participant type (e.g., youth, parent), age, sex, gender identity, ethnic and/or racial identity, socioeconomic position, and other sociocultural characteristics	Definition and status of migrant(s), country(ies) of origin and destination	Definition and type of pain examined, including factors and outcomes

**Table 2:** Sample table to contextualize studies within relevant geopolitical and international events.

	Study References (Author, Country) Grouped Above Their Respective Year of Publication										
Year	2015	2016	2017	2018	2019	2020	2021	2022	2023	2024	2025
	Significant international events potentially impacting migration, under each year										

## Data Availability

No new data were created or analyzed in this study. Data sharing is not applicable to this article.

## Abbreviations

The following abbreviations are used in this manuscript:

PCC	Population, concept, context
PRISMA-ScR	Preferred Reporting Items for Systematic Reviews and Meta-Analyses Extension for Scoping Reviews
PROGRESS-Plus	Place of residence, race/ethnicity/culture/language, occupation, gender/sex, religion, education, socioeconomic status, social capital
